# Supplementary material for: Patterns of Intron Gain and Loss in Fungi
Source: PLoS Biol. 2004 Nov 30;2(12):e422. doi: 10.1371/journal.pbio.0020422 (PMC532390; doi:10.1371/journal.pbio.0020422)
Supplement: Table S1 — Also available at http://genes.mit.edu/NielsenEtAl/. (4.3 MB ZIP). [file pbio.0020422.st001.zip › NielsenEtAl/html/1243.html]

AN5833.1.NCU01417.1.MG00689.1.FG10126.1


```
 CLUSTAL W (1.82) Multiple Sequence Alignments - Introns Inserted


Sequence 1: AN5833.1	694 aa
Sequence 2: FG10126.1	702 aa
Sequence 3: MG00689.1	700 aa
Sequence 4: NCU01417.1	709 aa
Alignment Length: 783 aa
Number Identitical Residues: 276 aa
Alignment Score (without introns) 15878


MG00689.1 	---------------------------------------------~--------MDSHPQ
NCU01417.1	MDSNTKTMSFVSKLATSTQNNLSIHIIASASSKLRPRDGCSLALH1RLGHITIDTMTHPQ
FG10126.1 	---------------------------------------------~---MPDNIEDYHVQ
AN5833.1  	---------------------------------------------~---------MTHPQ
          	                                                         * *

MG00689.1 	DDLHAHSLADPASFWGQQAEQLYWHKKPDAVLTESAKKVPDGDSEASHPHWEWFPGGEIS
NCU01417.1	DEAQHLSLTDPEGFWGHQAEQLHWHRKPSAVLKRTTKDLKLG---TSHSHWEWFPDGEIS
FG10126.1 	DEVLRYSLEDPEQFWRHQAEQLHWHKPFSSTIQLTQKTLRSG---ITHDSWEWFPDGEIS
AN5833.1  	QAVHAASLQNPEAFWSHHAQQLHWHKKPSRAIGRSTKTLASG---ASHESWSWFPDGEIS
          	:     ** :*  ** ::*:**:**:  . .:  : * :  *    :*  *.***.****

MG00689.1 	TCYNCLDRHVEKGNGDAPAIFYDSPVTGSKQRLSYKELLDEVALFAAVLRQEGVRKGDVV
NCU01417.1	TCYNCLDRHVLAGHGDQPAILYDSSVTNTKQRLTYKQLLAEVETFAGVLREEGVKKGDVV
FG10126.1 	TCYNCVDRHVLDGHGDSVAIYFDSPVTNTKEKYTYRQLLHEVETLAGALMEQGIGKGDVI
AN5833.1  	TTYNCVDRHVLNGNGDNVAIIWDSAVTGKKEKYTYRQLLDEVEVLAGVLREEGVKKGDVV
          	* ***:****  *:**  ** :**.**..*:: :*::** **  :*..* ::*: ****:

MG00689.1 	LVY------------------------M1PMIPAALIGILAINRLGAIHSVVFGGFASGA
NCU01417.1	LVYSQFTQLRLSRVSTEPATDSLPIYIV~PMVPATLIGILAINRLGAIHAVVFGGFASTA
FG10126.1 	MLY------------------------I1PTIPAAVIGILAANRLGAIHSIVFGGFAPFA
AN5833.1  	IIY------------------------M1PMIPAALIGALAVARLGAIHAAVFGGFAAKS
          	::*                        : * :**::** **  ******: ******. :

MG00689.1 	LAQRIDASRPVAILTASCGVEAGKGPIAYRNLVREAVAKSKWKPPKTIVWQREQLRWEPV
NCU01417.1	LAQRIEASRPVAILTASCGIEGNKGPVSYRAYIEEAISISSFKPPKTIIWQREQLVWRPI
FG10126.1 	LAQRIDSCHPVAILTASCGIDGNKPPIAYKPLVEEAIRLATHKPKDIIVWQRDQLRWN-I
AN5833.1  	LAQRIEAARPRAILTASCGIEGAKGPIAYRPLVEGAIEASSFKPEKVLIWQRDQLRWNNP
          	*****::.:* ********::. * *::*:  :. *:  :. ** . ::***:** *.  

MG00689.1 	RREDGERIWHKLVSSAKARKLPPAECVPVGSNDPIYIIYTSG~TTGLPKGVVRESGGHAV
NCU01417.1	KKTEGERDWQKLVKSARFRNIK-VECVPVRSADPIYIIYTSG~TTGRPKGVVRDSGGHAV
FG10126.1 	NTSAGQLDWKALTEKARSQWIK-ADCVPVKSTDPVYIIHTSG~TTGTPKGVLRDAGGHAV
AN5833.1  	DKLGGQRNWNRLVKSARMRGIR-AEPVPVRSTDGLYIIYTSG1TTGLPKGVVREAGGHAV
          	    *:  *: *...*: : :  .: *** * * :***:*** *** ****:*::*****

MG00689.1 	GLYLSINSKFGIHGPGDV0MACFSDIGWVVSHSYTLYGPLITGAATVLYEGKPVGTPDAG
NCU01417.1	GLHMSISYLFGIHGPGDV0APP--------------------------------------
FG10126.1 	GLHLSISYLFHITGPGCV~SFTASDIGWIVGHSYIIYGPLLTGAATVLYEGKPVGTPDAS
AN5833.1  	GLSLSIKYLFDIHGPGDT~MFCASDIGWVVGHSYILYAPLLVGATTVLFEGKPVGTPDAG
          	** :**.  * * *** .     :. .   . :    ..   .:::   .... .:..:.

MG00689.1 	AFWRIVEEYKVNSLFTAPTALRAIRK~ADPDNKLVEKYGRHGGLKSLRALFLAGERSEPA
NCU01417.1	---------------------RSSTK0EDPDNVFITEIGRRGGLKSLKALFLAGERSEPA
FG10126.1 	AFWRIVDEYKVNTMFTAPTALRAIRR~DDPYNQMFIDYGKRGALRSLRGLFLAGERSEPT
AN5833.1  	TFWRVVAEHKANVLFTAPTALRAIRK~EDPDNKHFEKVAGDNNLRHLRALFLAGERSEPS
          	:      . . .   ::.:: *:  :  ** *  . . .  . *: *:.**********:

MG00689.1 	IVEAYQEVLNKYAAPNAHVIDNWWSTESGSPISGIALVPQHGKDGKTCVRTQKTKPLSSK
NCU01417.1	IINMYQDLLRRYGAAGSQVVDNWWSSESGSPISGVALVPHAGKNR--KTNMKDHPPLPIK
FG10126.1 	LIEGYQKLMTQYCAENAQVVDNWWSTEVGSPITGRALVPHAGKDR--RTEVRGHQPPCLR
AN5833.1  	IVRAYQDLLTKHAARGALVVDNWWSSESGSPISGLALRSAVGRVPP-RSDEYDVAPLAIR
          	::. **.:: :: * .: *:*****:* ****:* ** .  *:            *   :

MG00689.1 	PGSAGKPMPGFDVR~VVNDEGEELPRGKMGNIVMAAPLAPTAFRTLWDDEPRFFRGYFKR
NCU01417.1	PGSAGKAMPGFDVR2VVDDEGNEVPRGNMGNIVLGLPLAPTAFRTLWGDEERFYKSYMIR
FG10126.1 	AGSAGKAMPGFDVR~IVDDEGVEVERESMGNIVLAMPLAPTGFRTLWQDEERFWKGYLKR
AN5833.1  	PGSAGLPMPGFDVR~VVDDEGNEVAQGTMGNIVMATPLAPTAFTRLFNDDERFYKGYLKR
          	.**** .******* :*:*** *: : .*****:. *****.*  *: *: **::.*: *

MG00689.1 	FNGLWVDTGDTGVVDEDGYVHIMSRSD1DIINVAAHRLST-~-G1TLEEAITSHPLVTEV
NCU01417.1	FNGKWVDTGDAGYVDQTGYIHIMARTD~DIINVAAH-----~-R1TLEQAVTSHPLVTEA
FG10126.1 	FDGKWLDTGDSGWIDRQGYIHVMARSD~DVLNVSAHRLSS-~-G1SIEQAIATHPRVVEV
AN5833.1  	FGGRWLDTGDAGMIDQDGYIHVMSRSD~DIINVAAHRFSTG1QG~SIEQAILSHPAIGEA
          	*.* *:****:* :*. **:*:*:*:* *::**:**  ::. .  ::*:*: :** : *.

MG00689.1 	CVVGIPDSLKGQMPFALVTTTP---NNEHVTDEQLLSEIQQLIRKQVGAIAALRGIIRGQ
NCU01417.1	CVVSVPDALKGQLPFAFVSTSD--GGADVKSDEQLFQDIQKLVRAQVGAIASLGGMIRGK
FG10126.1 	CIIGIPDSLKGQLPFAFVTLSGQVASDPAVPDQRVVKEIQQLVRGQVGAIASLGGIIQGK
AN5833.1  	SVVGIPDALKGHLPFAFITLKQSGGNSPARPSAELFNSVNRLVREQIGAIASLGGMIQGQ
          	.::.:**:***::***::: . . ..    .. .:...:::*:* *:****:* *:*:*:

MG00689.1 	GMIPKTRSGKTLRRVIRELFENARIGQADKEVTIPSTVEDAAVVEVARAKIKEYFEQQAK
NCU01417.1	GMIPKTRSGKTLRRVLRELLENAVHDEFEKPINIPSTVEDPSVVDVAREKIREYWHAKSG
FG10126.1 	CMIPKTRSGKMLRRVLKEMAENAIRGEFEKEVMVPSTIEDLATLDIARAKISEYFGQAET
AN5833.1  	GMIPKTRSGKTLRRVLRELVENGARGEFEKEVAVPPTVEDRGVVEVAREKVREYF-----
          	 ********* ****::*: **.  .: :* : :*.*:** ..:::** *: **:     

MG00689.1 	-GGGDRAPKAKL
NCU01417.1	-LHAAIEARAKL
FG10126.1 	RNAEDAMVKARL
AN5833.1  	-ESQSGSPKAKL
          	        :*:*
```
